# Online cognitive-behavioral based group interventions for adolescents with chronic illness and parents: study protocol of two multicenter randomized controlled trials

**DOI:** 10.1186/s12887-018-1216-6

**Published:** 2018-07-18

**Authors:** Miriam Douma, Linde Scholten, Heleen Maurice-Stam, Martha A. Grootenhuis

**Affiliations:** 1 Psychosocial Department, Emma Children’s Hospital, Amsterdam UMC, University of Amsterdam, Amsterdam, the Netherlands; 2 Princess Maxima Center for Pediatric Oncology, University Medical Center, Lundlaan 6, Postbus 85090, 3508 AB Utrecht, the Netherlands

**Keywords:** Chronic illness, Psychosocial functioning, Coping, Cognitive-behavioral therapy, Online psychosocial intervention, Group intervention, E-health, Adolescents, Parents

## Abstract

**Background:**

Adolescents with chronic illness (CI) and parents of a child with CI are at risk for psychosocial problems. Psychosocial group interventions may prevent these problems. With the use of cognitive-behavioral therapy, active coping strategies can be learned. Offering an intervention online eliminates logistic barriers (travel time and distance) and improves accessibility for participants. Aim of this study is to examine the effectiveness of two cognitive-behavioral based online group interventions, one for adolescents and one for parents: *Op Koers Online*. The approach is generic, which makes it easier for patients with rare illnesses to participate.

**Methods/design:**

This study conducts two separate multicenter randomized controlled trials. Participants are adolescents (12 to 18 years of age) with CI and parents of children (0 to 18 years of age) with CI. Participants are randomly allocated to the intervention group or the waitlist control group. Outcomes are measured with standardized questionnaires at baseline, after 8 (adolescents) or 6 (parents) weeks of treatment, and at 6- and 12-month follow-up period. Primary outcomes are psychosocial functioning (emotional and behavioral problems) and disease-related coping skills. Secondary outcomes for adolescents are self-esteem and quality of life. Secondary outcomes for parents are impact of the illness on family functioning, parental distress, social involvement and illness cognitions. The analyses will be performed according to the intention-to-treat principle. Primary and secondary outcomes will be assessed with linear mixed model analyses using SPSS.

**Discussion:**

These randomized controlled trials evaluate the effectiveness of two online group interventions improving psychosocial functioning in adolescents with CI and parents of children with CI. If proven effective, the intervention will be optimized and implemented in clinical practice.

**Trial registration:**

ISRCTN ISRCTN83623452. Registered 30 November 2017. Retrospectively registered.

## Background

Children and adolescents with a chronic illness (CI) have to face difficulties related to their illness, such as hospitalization, the use of medication, restrictions in activities and stressors related to the course of the illness and the future [1–3]. In the Netherlands, 14% of children and adolescents is growing up with a CI (for example diabetes, asthma or Cystic Fibrosis) [4]. Growing up with a CI influences psychosocial wellbeing and the development of cognitive and social skills [2, 5–7]. Especially during adolescence, with the formation of identity, self-image and self-esteem, a CI constitutes a major challenge [8, 9].

Research shows that pediatric CI influences psychosocial wellbeing in parents as well [10, 11]. Parents are predominantly responsible for managing the child’s illness. They are confronted with stressors about their child’s health as well as logistical and practical factors such as managing daily routines, relationships with other family members, the balance between family and work and possible financial problems [11, 12]. Therefore, parents are at risk for sorrow and psychosocial distress [10, 12]. Parents who face stress are less able to manage the child’s illness effectively [11]. To prevent and/or to reduce psychosocial problems in parents as well as adolescents, interventions focusing on how to cope with stressors caused by the CI are needed [13].

The disability-stress-coping model states that stressors related to the illness and psychosocial adjustment of children and mothers are moderated by coping strategies and cognitive appraisals [14]. Moreover, research has shown that effective use of coping skills increases adolescents’ medical compliance, improves their psychosocial functioning [2, 15–20] and reduces distress and anxiety in parents [21, 22]. Results on the effectiveness of cognitive-behavioral based psychosocial group interventions to learn children and adolescents how to use more effective coping skills are promising [23–25]. Research shows that including parents in a psychosocial intervention for children with chronic pain is effective in reducing child’s pain [22]. There is some evidence of effectiveness of interventions focusing on parents themselves: coping support interventions reduce parental psychological problems during acute hospitalization [21] and problem solving therapy for parents improves parental mental health [22]. However, little is known about the effectiveness of cognitive-behavioral based psychosocial group interventions for parents focusing on themselves.

In 2003, the face-to-face cognitive-behavioral based group intervention *Op Koers* (English: *On Track)* for children and adolescent with CI was developed in the Emma Children’s Hospital (Academic Medical Center Amsterdam). Over the years, the program was expanded with courses for siblings and parents, and a similar *Op Koers* program for pediatric oncology patients. Goal of *Op Koers* is to prevent and/or to reduce psychosocial problems of participants by teaching active coping skills with the use of cognitive-behavioral therapy (CBT) techniques [26]. The approach is generic, which means that patients with every type of CI can participate. This has the benefit of giving more patients at once the opportunity to participate and to include patients with rare illnesses in group interventions [23, 26]. Sharing experiences with others in a similar situation had been associated with a decrease of distress and improvement of social health [23, 27–29] and is therefore an important part of *Op Koers*. There have been different studies about this intervention program [26, 30, 31]. Part of the research has been an RCT on the efficacy of *Op Koers* for children and adolescents with CI. This study showed positive short- (half year) and long-term (one year) effects on the use of coping skills and psychosocial functioning. For children and adolescents, there was an additional positive effect of parental involvement, especially on long-term and in social-emotional vulnerable children [32–34].

The face-to-face setting of *Op Koers* requires participants to regularly come to the hospital, in addition to other medical appointments. An online intervention eliminates logistical barriers such as travel time and distance [35, 36] which makes the intervention more easily accessible [35, 37, 38]. Participating in an intervention online connects to the digital environment in which people live nowadays. Besides, an online environment without use of a webcam increases anonymity: appearance plays no role and this might make it easier to talk about problems [38–40]. Therefore, *Op Koers* was translated into an online intervention: *Op Koers Online*.

First, the intervention for survivors of childhood cancer was developed. A pilot study on the feasibility of *Op Koers Online Oncology* for adolescent survivors showed promising results [39]*.* The intervention was optimized based on feedback from participants and course leaders (for example: expanding the sessions from six to eight). After that, *Op Koers Online* for adolescents with CI was developed. Similar to the face-to-face intervention, goal is to prevent and/or reduce psychosocial problems by teaching the use of active coping skills with CBT techniques. Sharing experiences with other chronically ill adolescents is an important part of the intervention. First pilot results on the feasibility and potential effectiveness of *Op Koers Online* for adolescents with CI are promising (Douma et al.: Feasibility and effectiveness of an online cognitive-behavioral based group intervention for adolescents with chronic illness, submitted).

For parents, most existing interventions focus on the child’s functioning [13, 22]. The same applies to *Op Koers* face-to-face for parents, where participating parents learn what their child learns and how to support their child in implementing coping skills in daily life [32, 34]. However, research suggests the need of emotional, informational and peer support for parents [41, 42]. For the development of *Op Koers Online* for parents, the Emma Children’s Hospital (Academic Medical Center, Amsterdam) conducted a survey among parents on their specific wishes and needs. The need for an intervention focusing on parental functioning instead of focusing only on the child emerged from the survey. Based on the results of this survey, *Op Koers Online* for parents of children with CI was designed.

This paper describes the rationale and the design of two separate multi-center randomized controlled trails aimed to assess the extent to which *Op Koers Online* is effective in preventing and/or reducing psychosocial problems (emotional/behavioral problems and quality of life) and improving the use of disease-related coping skills in adolescents with CI (12–18 years) and in parents of children and adolescents (0–18 years) with CI.

## Methods

### Procedure

**Fig. 1:**
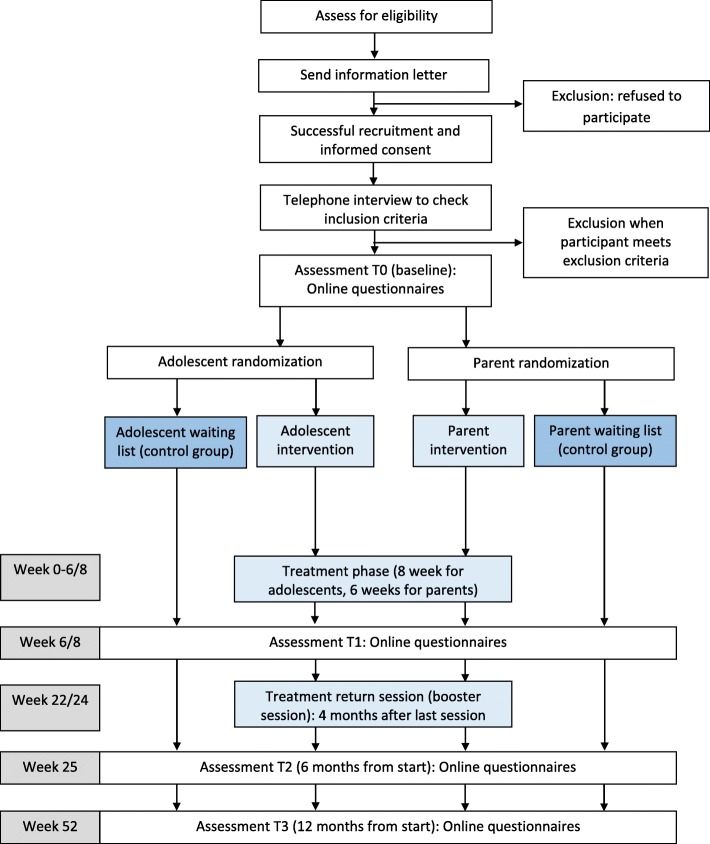
Study procedure in flow diagram

Figure 1 shows the different phases of the study procedure. There is one coordinating hospital (Emma Children’s Hospital, Academic Medical Center, Amsterdam) and eight participating hospitals across the Netherlands (one academic, seven non-academic). The researcher of the coordinating hospital coordinates overall recruitment and administers inclusion of all participants. Local recruitment is coordinated by local investigators of each participating hospital. Adolescents and parents from the outpatient clinics from the nine hospitals receive an information letter from their pediatrician. To improve inclusion of adolescents and parents for the study, we asked permission from the Medical Ethical Committee (METC) to make the procedure open accessible and permission was obtained. Besides the information letters, pamphlets are available in the participating hospitals and other interested hospitals (approached randomly by the coordinating researcher). Recruitment is done via internet (websites and social media) and via patient associations.

Interested adolescents and parents are asked to return the application form added to the pamphlet or to send an e-mail. A telephonic interview is used to screen inclusion criteria, discuss the information about the intervention and the study and to discuss the informed consent. Potential participants can ask questions and get one week to overthink participation. When willing to participate, an informed consent form is sent to the participant to sign and return. As soon as the informed consent form is signed by the researcher as well, the researcher registers the participant online (http://www.opkoersonline.nl). Participants receive an e-mail with a link to create personal login codes, with which they can login to the secured website.

Every registered participant is in the virtual ‘waiting room’ until randomization. They are informed about the result of the randomization by e-mail. When randomized in the intervention group, the researcher calls the participant to determine the dates of the intervention. At four time-points, all participants and parents of participating adolescents are invited to complete questionnaires via an e-mail with a personal link to the questionnaire. Total duration time for completion is estimated at 45 min for adolescents and parents and 30 min for the parents of participating adolescents. After completing all assessments, participants receive a financial reward (€20 voucher for an online book/game store).

### Interventions

**Fig. 2:**
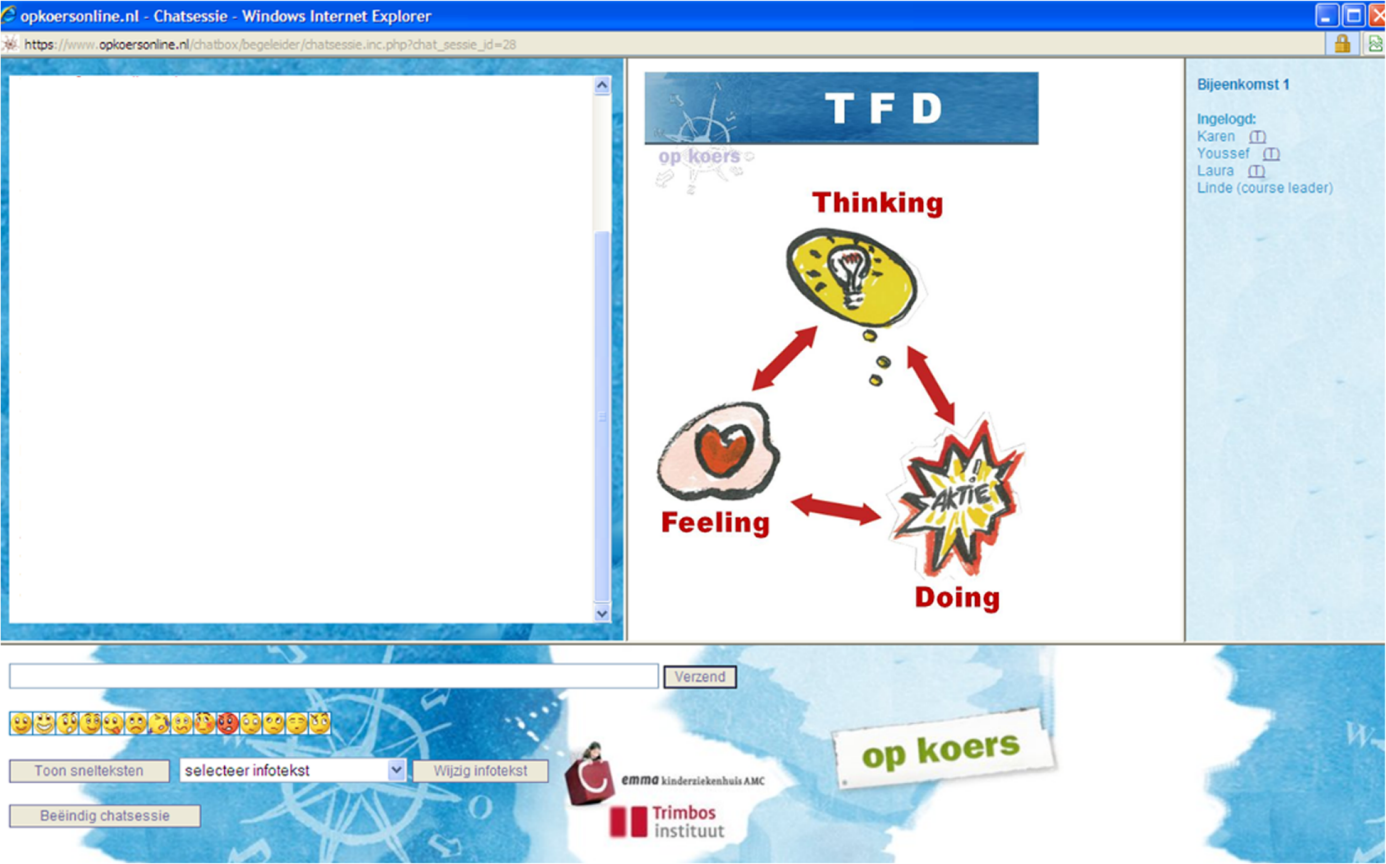
Chat room session (left screen: chat; right screen: information; strip right: list of present participants)

The interventions consist of eight (for adolescents) or six (for parents) weekly sessions of 90 min, which take place in a secured chatroom (Fig. 2) with groups of three to six participants. The interventions are guided by two course leaders, one specialized health-care psychologist and one co-therapist (mostly a psychological assistant), who are trained and use a detailed manual. The training consists of three parts: 1) teaching the main principles of cognitive-behavioral group therapy and the history of the *Op Koers* courses, 2) giving more specific information on the procedures and goals related to the different sessions using examples from former chat sessions and the extensive manual for psychologists, 3) practicing in online subgroups. To ensure treatment integrity, the researcher of the coordinating hospital randomly checks the chats of participating hospitals with the manual. All participants and course leaders log on at the same time every week. Participants can log on to the homework site to view the intervention material (information sheets and videos), submit homework before every session and view additional information. Four months after the last session, there is a booster session.

Central in the interventions is the Thinking-Feeling-Doing model (TFD model). With this model, course leaders teach participants the relationship between what people think, feel and how they act, and how they can influence their thoughts feelings and behaviors. Every intervention group starts the first session with an extensive acquaintance (questions such as: who are you, what do you do, which illness do you/does your child have, what are your expectations of the course, etc.) to create a feeling of safety within the group and in the chat box. No webcams are used in the interventions.

### ‘Op Koers Online’ for adolescents

The aim of the intervention for adolescents (12 to 18 years) is to prevent and/or to reduce psychosocial problems, by teaching the use of active coping strategies. These strategies are taught by recognizing negative thoughts and transforming them into more positive and proactive ones, with the use of CBT techniques (TFD-model) [25, 43, 44].

Learning goals of the adolescent intervention are increasing the use of five coping skills taught with the CBT techniques (e.g. relaxation, cognitive restructuring and open communication) [43, 45, 46]: 1) information seeking and giving about the illness, 2) use of relaxation during stressful (medical) situations, 3) increase knowledge of self-management and medical compliance, 4) improvement of social competence and 5) positive thinking [26, 34]. See Table 1 for learning goals and corresponding instructions and reinforcement techniques. Each coping skill is taught during one specific session, but elements of the coping skills are also addressed in the subsequent sessions. The skills are taught by psycho-education (e.g. video’s, group discussions), through exercises (e.g. virtual board games) and homework assignments (e.g. practicing relaxation exercise in daily life).

**Table 1:** Learning goals and examples of learning activities of *Op Koers* Online for adolescents and parents

		Learning goals						
		Information seeking and giving about the illness	Use of relaxation during stressfull situations	Increase knowledge of self-management and compliance	Enhancement of social competence	Positive thinking	Positive parenting	Open communication & seeking and accepting support
Examples of learning activities of the adolescent intervention	Instruction/modelling	Education about sources of information	Relaxation exercise (MP3)	Group discussion about own treatment and (non-)compliance	Video and group discussion: how and what do you tell others about your illness	Thinking-Feeling-Doing game		
	Reinforcement/practice (homework)	Write down questions you have, and look for answers	Practice the relaxation exercise	Write down situations for non-compliance and how to improve compliance	Think of what CAN you do (instead of CANNOT) and write down your story for the other group members	Write down positive adjustments for negative thoughts		
Examples of learning activities of the adolescent intervention	Instruction/modelling		Group discussion: take care of yourself and relaxation exercise (MP3)	Group discussion: medical treatment and compliance		Discussing the Thinking-Feeling-Doing model	Group discussion: how to divide your attention between different family members	Group discussion: what kind of support do you receive, like and want (practical/ emotional)
	Reinforcement/practice (homework)		Practice the relaxation exercise	Together with your child (when possible): make a list concerning medical treatment and discuss (non-)compliance		Learning about negative thoughts and how to replace them for positive ones	Make a list of activities to do with different family members (apart)	Think of people who could help and support you and write down what you would ask them

### ‘Op Koers Online’ for parents

Aim of the intervention for parents is also to prevent and/or to reduce psychosocial problems by teaching the use of active coping strategies. Strategies to help parents focusing on elements they think are important in life, and to act conform these elements, are taught with the use of CBT techniques and Acceptance and Commitment Therapy (ACT). ACT, part of CBT, is an intervention strategy to learn participants how to accept a new situation (such as: having a child with CI) and to establish new routines. Goal is to increase or create psychological flexibility. This is done with relaxation exercises and reflection which helps participants to remind and recognize what barriers they face in achieving goals and living consistent with their values, and how to adjust behavior in these situations [47–49]. There is growing evidence for the effectiveness of ACT [47, 50–52].

Learning goals of the parent intervention are increasing the use of five coping skills taught with CBT and ACT techniques: 1) use of relaxation during stressful situations, 2) increase knowledge of self-management and compliance of their child, 3) positive thinking, 4) positive parenting and 5) open communication about the illness and seeking and accepting support. See Table 1 for learning goals and corresponding instructions and reinforcement techniques.

Every session has a subject. However, specific content of each session is determined by parents: what they want to discuss. Subjects are: the parent (e.g. taking care of yourself), the family (e.g. positive parenting), the hospital (e.g. child’s compliance), extended family and friends (e.g. seeking and accepting support), and daily life (e.g. work, school; open communication). Participants are asked to answer questions concerning the subject of the session (for example the following question about the subject ‘the family’: “How does the illness affect your child his/her siblings/you and your (ex-)partner?”) and to react on each other (giving tips, asking questions, sharing experiences). The questions are displayed in the right screen of the chat box. An important part is sharing experiences with other group members and giving and receiving social/emotional support. Compared to the intervention for adolescents, the intervention for parents is less protocolled. There is more room for personal input and (spontaneous) group discussions, and there are less video’s, games and exercises during the sessions.

### Inclusion and exclusion criteria

Adolescents between 12 and 18 years old with CI, and parents of children between 0 and 18 years old with CI are included. The term CI refers to an illness that requires at least six months of continuous medical care, permanent life style changes and continuous behavioral adaptation to the unpredictable course of the illness [4]. Participants (for parents: their child) have to be treated by a pediatric specialist in a pediatric hospital in the Netherlands. Adolescents and parents of participating adolescents should be able to fill out Dutch questionnaires and to follow the chat intervention in Dutch. A computer or tablet with internet connection to enter the website and chat box is necessary. Adolescents and parents with severe learning difficulties are excluded from the intervention. For them, an adapted or individual program might fit better to their individual cognitive needs.

### Study design

The current study consists of two separate multi-center randomized controlled trails: one for adolescents and one for parents. Both trials have two conditions: the intervention group (*Op Koers* Online) and the waitlist control group. An adolescent and a parent can both participate, but this is not required. When both parents want to apply for the study, they can participate separately. Participants assigned to the waitlist control group receive care-as-usual and are not prevented to seek individual psychosocial treatment. If a participant needs psychosocial care, this will be approved. When participants from either the intervention or the control group receive (additional) psychological treatment during the study period, it will be extensively documented and controlled for in the analyses. When the study is finished, participants from the waitlist group have the opportunity to participate in the intervention.

Assessment of outcome measures takes place with online questionnaires at baseline (before randomization; T0), directly after the intervention period (eight weeks for adolescents, six weeks for parents; T1), six months (T2) and twelve months (T3) after baseline. For adolescents participating in the study, one of their parents is asked to complete questionnaires as well.

This study was approved by the METC of the Academic Medical Center Amsterdam and of the eight participating hospitals.

### Randomization

With an average of five participants per intervention group, a total of ten intervention groups for both adolescents and parents will be given. Interventions are organized at different time points (in four to six cohorts, dependent on inclusion rates). In each cohort, participants are randomly allocated to the conditions resulting in an equal number of participants in the intervention and waitlist control condition. The randomization is carried out by an independent IT worker from a company for e-health development who administers the website for *Op Koers Online*, using block randomization software.

### Sample size

Earlier studies on the effectiveness of *Op Koers* and comparable effect studies showed effects of medium size [32, 53]. Based on a design with four repeated measurements and a within subject correlation of .5, 84 adolescents and 84 parents are needed – 42 in each condition – to detect an intervention effect of medium size (d = .05) over time, at a two-sided .05 significance level and 80% power. Taking into account a dropout of 15% over time, 96 adolescents and 96 parents are needed to achieve the intended power.

### Outcome measures

#### Questionnaires

The outcome measures will be assessed by standardized questionnaires with good psychometric qualities and available normative data (Table 2) [26, 54–63]. To assess participants’ satisfaction with the intervention, content, design and course leaders, participants in the intervention group complete an evaluation questionnaire at the end of the intervention period (T1).

**Table 2:** Primary/secondary outcome measures, measurements and informant for the adolescent and parent intervention

	Measurements	Informant
Primary outcome measures		
Adolescents		
Psychosocial functioning	Child Behavior Checklist (CBCL) and Youth Self Report (YSR)	Parent and adolescent
Disease-related coping skills	*Op Koers* Questionnaire (OKQ)	Adolescent
Parents		
Psychosocial functioning	Hospital Anxiety and Depression Scale (HADS)	Parent
Disease-related coping skills	*Op Koers* Questionnaire (OKQ)	Parent
Secondary outcome measures		
Adolescents		
Self-esteem	Perceived Competence Scale for Adolescents (CBSA)	Adolescent
Quality of Life	Pediatric Quality of Like Inventory - self report (PedsQL)	Adolescent
Parents		
Impact of the illness on family functioning	Pediatric Quality of Life Inventory - Family Impact Module (PedsQL-FIM)	Parent
Parental distress	Distress Thermometer for Parents (DTP)	Parent
Social involvement	Inventory Social Involvement (ISI)	Parent
Illness cognitions	Illness Cognition Questionnaire for Parents (ISQ)	Parent

### Statistical analyses

Analyses will be performed according to the intention-to-treat principle. Primary and secondary outcomes will be assessed with linear mixed model analyses using SPSS. The intervention is qualified as effective if the intervention group improved more over time on one of the primary outcomes than the control group, at a significance level of 0.05 and at small to medium effect size d [0.2–0.5].

## Discussion

This paper outlines the study protocol for two multicenter randomized controlled trials on the effects of two cognitive-behavioral based online group interventions: one for adolescents with CI and one for parents of children and adolescents with CI. Earlier studies showed that psychological interventions for children and adolescents with CI, and for parents, can improve psychosocial functioning [22, 23, 32]. Also, studies on effectiveness of online interventions showed promising results [40, 64–66]. Online interventions are easily accessible and, when not using a webcam, anonymous [38–40]. These factors can increase possibility and willingness from participants to apply for a psychosocial intervention. There is a lack of evidence-based online group interventions for adolescents with CI and for parents. Studies in this field are limited. Therefore, this study is unique in focusing on an online cognitive-behavioral group intervention for these populations.

This study has several strong points. First, participation in the intervention and the study are completely online, which eliminates logistical barriers for participants and therefore keeps drop-out rate low. Second, we include nationwide with focus on a heterogeneous group of different medical chronic diagnosis. This way, is easier to achieve a relatively large study sample, which is beneficial for the statistical power. Third strong point is that participants in the intervention group can be divided over treatment groups independent of the hospital, which benefits the feasibility of the study (it is easier to create intervention groups on different time points, this will overcome drop-out due to availability). Finally, the relatively long term follow-up period promotes stronger long-term results.

Some vulnerabilities have also to be taken into account. First, since recruitment of adolescents for health studies is challenging [67, 68], the intervention for adolescents is at risk for recruitment problems or delay. This could be a threat to the inclusion rates and the statistical power of the study. Second, due to the relatively long follow-up period it is possible that participants will seek other psychosocial support in the study period. This could bias the results. Lastly, since we include nationwide, it is impossible to identify the approached group and to determine non-response.

In conclusion, adolescents with CI and parents of children and adolescents with CI are at risk for developing psychosocial problems. Easily accessible online evidence-based interventions are needed. This study aims to contribute to research on effective interventions for adolescents with CI and parents of children and adolescents with CI by investigating two separate group interventions, for adolescents and for parents. If this study shows significant effects of the interventions on improving psychosocial wellbeing and disease related coping skills in adolescents and/or parents, *Op Koers Online* will be implemented in clinical practice.

## Abbreviations

ACT: Acceptance and Commitment Therapy
CBT: Cognitive-behavioral therapy
CI: Chronic illness
METC: Medical Ethical Committee
TFD model: Thinking-Feeling-Doing model
